# Donor-derived 47, XXY in an unrelated cord blood transplant recipient

**DOI:** 10.1186/2193-1801-3-72

**Published:** 2014-02-06

**Authors:** Kuniki Kawaguchi, Takayuki Nakamura, Masayuki Nohara, Satoko Koteda, Kei Nomura, Satoshi Morishige, Eijiro Oku, Rie Imamura, Fumihiko Mouri, Ritsuko Seki, Koichi Osaki, Michitoshi Hashiguchi, Kohji Yoshimoto, Koji Nagafuji, Takashi Okamura

**Affiliations:** Department of Medicine, Division of Hematology and Oncology, Kurume University School of Medicine, 67 Asahi-machi, Kurume, 830-0011 Japan

**Keywords:** Chromosomal abnormalities, Donor-derived, Cord blood, Hematopoietic stem cell, Transplantation

## Abstract

A 65-year-old Japanese male with therapy-related myelodysplastic syndrome was admitted for unrelated cord blood transplantation. A cord blood unit from a male donor was obtained from the Japan Cord Blood Bank Network. The patient then received a conditioning regimen consisting of fludarabine, intravenous busulfan, and total body irradiation. Successful engraftment was obtained. The bone marrow examination on day 28 revealed trilineage engraftment, and chimerism analysis by variable number of tandem repeat polymerase chain reaction confirmed complete donor chimerism. At that time, conventional cytogenetics of the bone marrow aspirate showed 20 out of 20 metaphases with the 47, XXY karyotype characteristic of Klinefelter syndrome. Klinefelter syndrome is the most common genetic cause of human male infertility with a reported prevalence of 0.1–0.2% in the general population. In Japan Cord Blood Bank Network, there is no informed consent from parents about the possibility that post-unrelated cord blood transplantation patient evaluation may reveal donor-origin inherited diseases including cytogenetic abnormality. It is desirable to have opportunities in Japan discussing whether parents will be notified of the possibility that post-unrelated cord blood transplantation evaluation may reveal donor-derived illness incidentally.

## Background

The use of unrelated cord blood (CB) as a source of hematopoietic stem cells has been increasing in Japan. Indeed, more than a thousand unrelated cord blood transplantations (UCBTs) are performed annually in Japan (Japanese Cord Blood Bank Network, 2014). One of the distinct disadvantages of cord blood unit use is the lack of a significant clinical history from the donor. To compensate for this lack, the Japanese Cord Blood Bank Network screens the donor’s parents for genetic diseases, screens placental and maternal blood for infectious diseases, and performs blood and HLA typing. In addition, the Japanese Cord Blood Bank Network has a mandatory quarantine period of 6 months so that units cannot be used until a minimal amount of adverse medical event data on the donor can be obtained (Moore et al. 2005).

The informed consent for cord blood donation is obtained from the parents and of course not from the fetus or infant. To confirm engraftment after UCBT and evaluate the status of the primary disease, cytogenetic analysis of bone marrow is performed routinely. These analyses identify indirectly the karyotype of donor cells. Here, we report the case of a UCBT recipient whose post-transplant karyotype analysis revealed 47, XXY.

## Case description

A 65-year-old Japanese male was admitted to our hospital for UCBT in 2012.

In 2003, he was diagnosed with follicular lymphoma stage IVA, and treated with rituximab-containing chemotherapy resulting in a first complete remission (CR). His first relapse occurred in 2004 and treatment consisted of three courses of rituximab and the CHASE regimen (Ogura et al. 2003). His second relapse occurred in 2007 and treatment with rituximab, the CHASE regimen, and then autologous peripheral blood stem cell transplantation led to a third CR (Kamezaki et al. 2007). His third relapse occurred in 2009, and was treated with ^90^yttrium-ibritumomab-tiuxetan, obtaining a fourth CR.

**Figure 1:**
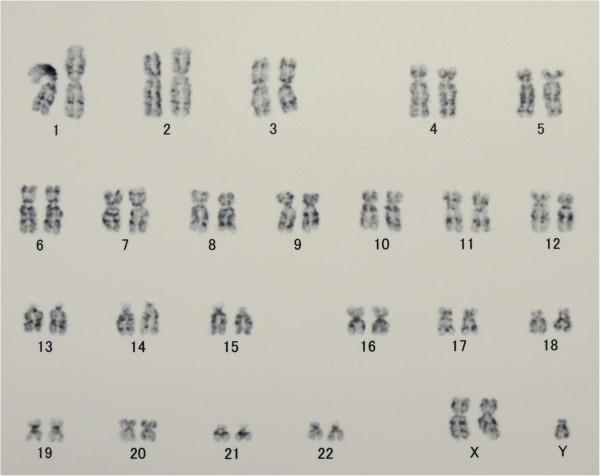
**The bone marrow examination on day 28 revealed trilineage engraftment and conventional cytogenetics of the bone marrow aspirate showed 20 out of 20 metaphases with 47, XXY karyotype.**

In 2012, routine blood examination revealed immature cells in the peripheral blood, and bone marrow examination confirmed the diagnosis of therapy-related myelodysplastic syndrome RAEB-1 with the abnormal karyotype of 45, XY, del5q, del7q, del12p, -13. His International Prognostic Scoring System (IPSS) score was Int-2 (Greenberg et al. 1997). A cord blood unit from a male donor was obtained from the Japan Cord Blood Bank Network, was mismatched at 2 HLA loci, contained 1.93 × 10^7^/kg of nucleated cells, and contained 0.62 × 10^5^/kg of CD34-positive cells. The patient then received a conditioning regimen consisting of fludarabine (30 mg/m^2^ on days –7 to –2), intravenous busulfan (3.2 mg/kg on days –6 to –3), and total body irradiation (4 Gy on day –2) (Takagi et al. 2010) as well as graft-versus-host disease (GVHD) prophylaxis consisting of intravenous tacrolimus (0.02 mg/kg/day) beginning on day –1 and short-term methotrexate on days 1, 3, and 6 (5 mg/m^2^). Engraftment was obtained. A neutrophil count of > 0.5 × 10^3^/L and a platelet count of > 20 × 10^3^/L were achieved on days 14 and 42, respectively. He developed acute GVHD grade II of the gut and was successfully treated with hydrocortisone. The bone marrow examination on day 28 revealed trilineage engraftment, and chimerism analysis by variable number of tandem repeat using the AmpFLSTR® SGM Plus® PCR Amplification Kit (Applied Biosystems) confirmed the establishment of complete donor chimerism. At that time, conventional cytogenetics of the bone marrow aspirate showed 20 out of 20 metaphases with 47, XXY (Figure 1). On day 130, peripheral blood examination revealed emergence of blasts, and on day 137, bone marrow examination showed recurrence of myelodysplastic syndrome. At that time, chimerism analysis revealed mixed chimerism of 64.4% donor origin, and conventional cytogenetics of the bone marrow aspirate showed 14 out of 16 metaphases with 47, XXY, and 2 out of 16 metaphases with 45, XY, -3, del5q, add7q, del12p, -13, + mar. Gradually cytopenia worsened and the patient was treated conservatively.

## Discussion

Klinefelter syndrome was first described in 1942 as an endocrine disorder characterized by small firm testes, gynecomastia, hypogonadism, and higher than normal concentrations of follicle-stimulating hormone (FSH) (Klinefelter et al. 1942). About 80% of cases are due to the congenital numerical chromosome aberration 47, XXY (Lanfranco et al. 2004). Klinefelter syndrome is the most common genetic cause of human male infertility with a reported prevalence of 0.1–0.2% in the general population, but many cases remain undiagnosed because of substantial variation in clinical presentation and insufficient professional awareness of the syndrome itself. Abramsky and Chapple calculated that 10% of expected cases are identified prenatally and 26% are diagnosed in childhood or adult life because of hypogonadism, gynecomastia, or infertility, leaving 64% undiagnosed (Abramsky and Chapple 1997). Indeed, Halaburda et al. reported diagnosis of Klinefelter syndrome in the donor after transplantation of allogeneic peripheral blood stem cells into a 35-year-old recipient with acute myelogenous leukemia from his 37-year-old HLA-identical brother (Halaburda et al. 2000). In Japan, more than a thousand UCBTs have been performed annually, and the prevalence of Klinefelter syndrome is supposed to be 0.1–0.2%. A cytogenetic survey conducted in Japan on 14,835 liveborn infants (7608 males and 7227 females) showed that 93 infants (6.27 per 1000) had a major chromosome abnormality, and of these, seven male (0.92 per 1000 male) infants had a 47, XXY karyotype. Thus, UCBT from male donors with Klinefelter syndrome like ours seems to happen continuously. In the Japanese Cord Blood Network, mothers must provide informed consent whether or not they want to know their infectious disease screening results (Japanese Cord Blood Bank Network, 2014). However, early in the course of their pregnancies, most mothers have been checked for infectious diseases as part of a routine screening program in Japan. Notably, parents who provide informed consent are not told about the possibility that donor-origin inherited diseases including cytogenetic abnormalities may develop post-UCBT. The prognosis of these inherited diseases may range from very mild to dismal. Among them, Klinefelter syndrome is a disease that with early recognition and hormonal treatment of the disorder can be managed, and quality of life can be substantially improved (Mehta and Paduch 2012). Post-UCBT, cytogenetic analysis of bone marrow is performed routinely. These analyses identify indirectly the karyotype of donor cells. Various types of constitutional abnormalities of karyotype have been reported to be transmitted after allogeneic hematopoietic stem cell transplantation as shown in Table 1 (Graze et al. 1977; Becher et al. 1986; Kuffel et al. 1991; Barquinero et al. 1995; Halaburda et al. 2000; Moore et al. 2005; Manola et al. 2006; Ismail et al. 2007; Balci et al. 2008; Frey et al. 2008; Consoli et al. 2011). All donors with Down syndrome were identified before stem cell donations as the manifestations of Down syndrome were evident. On the contrary, most donors with numerical abnormalities of sex chromosomes were diagnosed after transplantation; it means the manifestations of numerical abnormalities of sex chromosomes were varying much and post-transplant chromosomal analysis of recipients revealed these numerical abnormalities. In the setting of UCBT, as far as we know, only one case of the donor-derived 47, XXX karyotype has been reported in the literature (Moore et al. 2005). The Netcord and Foundation for Accreditation of Cellular Therapy (NETCORD-FACT) international standards for cord blood collection, banking, and release for administration recommends in the informed consent section that the cord blood bank “maintain linkage for the purpose of notifying the infant donor’s mother or family and/or her physician of communicable or genetic diseases, whenever possible” (International Standards for Cord Blood Collection, Banking, and Release for Administration 2013). On the contrary, in Japan Cord Blood Banking Network, there is no informed consent from parents about the possibility that post-UCBT patient evaluation may reveal donor-origin inherited diseases including cytogenetic abnormality. It is a matter of great delicacy. Some parents may want to know their child’s abnormality, and others may not (Sugarman et al. 2002). It is desirable to have opportunities in Japan discussing whether parents will be notified of the possibility that post-UCBT evaluation may reveal donor-derived illness incidentally.

**Table 1:** **Reported cases of donor-derived chromosomal abnormalities in recipients with allogeneic stem cell transplantations**

Type of transplantation	Donor type	Donor age	Donor sex	Chromosomal abnormalities	Timing of the diagnosis	References
BMT	R	29	M	46, XY, t(18q+;22q-)	Before transplant	Graze et al. 1977
BMT	R	28	F	47, XXX	After transplant	Becher et al. 1986
BMT	R	19	F	45, XX; rob t(14;14)	After transplant	Becher et al. 1986
BMT	R	NA	NA	47, XXY	NA	Kuffel et al. 1991
BMT	R	NA	NA	47, XXY	NA	Kuffel et al. 1991
BMT	R	NA	NA	45, X/46, XX	NA	Kuffel et al. 1991
BMT	R	NA	NA	46, XX/47, XX, +mar	NA	Kuffel et al. 1991
BMT	R	13	F	Down syndrome	Before transplant	Barquinero et al. 1995
BMT	R	26	M	Down syndrome	Before transplant	Barquinero et al. 1995
BMT	R	23	F	Down syndrome	Before transplant	Barquinero et al. 1995
BMT	R	17	F	Down syndrome	Before transplant	Barquinero et al. 1995
BMT	R	25	F	47, XXX	After transplant	Barquinero et al. 1995
BMT	R	48	F	47, XXX	After transplant	Barquinero et al. 1995
PBSCT	R	37	M	47, XXY	After transplant	Halaburda et al. 2000
CBT	U	0	F	47, XXX	After transplant	Moore et al. 2005
NA	R	2.5	F	45, X/46, XX	After transplant	Manola et al. 2006
NA	R	54	F	45, X/46, XX	After transplant	Manola et al. 2006
BMT	R	23	M	46, XX/46, XX, dic(Y;22)(p11.2;p11.2)	After transplant	Ismail et al. 2007
BMT & CBT	R	11 month	M	47, XXY	Before transplant	Balci et al. 2008
BMT	U	26	M	47, XY, +8	After transplant	Frey et al. 2008
BMT	U	NA	M	45, XY, der(14;21)(q10;q10)	After transplant	Consoli et al. 2011

*Abbreviations:* BMT: bone marrow transplantation, PBSCT: peripheral blood stem cell transplantation, CBT; cord blood transplantation R: related, U: unrelated, NA: not available, M: male, F: female.

## Conclusions

We described donor-derived cytogenetic abnormalities of 47, XXY in the recipient of UCBT. It is desirable to have opportunities in Japan discussing whether parents will be notified of the possibility that post-UCBT evaluation may reveal donor-derived illness incidentally.

### Consent

Written informed consent was obtained from the patient for the publication of this report and any accompanying images.
